# Elevated Opioid Growth Factor Alters the Limbus in Type 1 Diabetic Rats

**DOI:** 10.33696/diabetes.4.054

**Published:** 2023

**Authors:** Patricia J. McLaughlin, Joseph W. Sassani, David Diaz, Ian S. Zagon

**Affiliations:** 1 Department of Neural and Behavioral Sciences, Penn State University College of Medicine, Hershey, Pennsylvania 17033, USA; 2 Department of Ophthalmology, Penn State University College of Medicine, Hershey, Pennsylvania 17033, USA

**Keywords:** Type 1 diabetes, Limbus, Naltrexone therapy, Opioid growth factor, Ki-67, CK15

## Abstract

Ocular surface complications occur in more than 50% of individuals diagnosed with diabetes. The financial and health-related burden of diabetes is increasing annually. Several major ocular complications associated with diabetes involve the limbus. The vascular limbus, adjacent to the avascular cornea, is the source of circulating growth factors, elevated glucose, and cytokines for the cornea. The Opioid Growth Factor (OGF) - Opioid OGF Receptor (OGFr) axis is comprised of its effector peptide, OGF, [Met^5^]-enkephalin and the nuclear-associated receptor, OGFr, and has been demonstrated to be dysfunctional in diabetes with elevated serum and tissue levels of the inhibitory growth factor OGF recorded in corneal tissue. Little is known regarding the impact of OGF-OGFr axis dysregulation in diabetes on the functioning of the limbus constituents in support of corneal homeostasis. Adult male and female Sprague-Dawley rats were rendered hyperglycemic through intraperitoneal injections of streptozotocin (T1D); a subset of T1D rats received topical naltrexone (NTX) applied to the cornea and limbus daily for 8 weeks. At 4 and/or 8 weeks of hyperglycemia, different cohorts of animals were euthanized, eyes removed and processed for assessment of limbal morphology, expression of OGF, OGFr, cytokeratin 15, a marker for limbal cells, and Ki-67, a marker of proliferation. Limbal epithelial morphology (cell diameter, packing density) was altered in T1D male and female rats. OGF and OGFr were overexpressed in the limbus and CK15 expression was decreased, relative to normal control rats of the same sex. Blockade of the OGF- OGFr axis with NTX reversed limbal epithelial cell defects, and reduced OGF limbal tissue levels to those recorded in non-diabetic rats. In summary, OGF-OGFr axis dysregulation was observed in the limbus of T1D rats, contributing to the altered limbal morphology and delayed corneal surface healing observed in diabetic animals.

## Introduction

Reports from the Center for Disease Control and Prevention in early 2022 estimated that approximately 37 million people in the United States have diagnosed or undiagnosed diabetes and an additional 96 million have prediabetes [1]. Worldwide, nearly 11% of the global population is projected to be diagnosed with diabetes by 2045 [2]. The incidence of newly diagnosed diabetes in the United States is highest among Hispanics, Native American/Alaska Natives, and non-Hispanic Blacks making diabetes an important medical concern for the underserved [3]. The financial burden of diabetes and the treatment of its complications are estimated to approach $327 billion in the United States by 2045 [4]. Diabetic ocular surface complications include diminished tear production, abnormalities in corneal sensitivity, and delayed corneal epithelial wound healing are chronic and often more debilitating that the disease itself [5-7]. These complications may result from alterations in the limbus. Located adjacent to the cornea, the limbus plays a role in the healing of the corneal surface by providing limbal epithelial stem cells that undergo mitosis and ultimately provide terminally differentiated corneal epithelial cells [8,9]. Interactions between the Opioid Growth Factor (OGF) – OGF receptor (OGFr) pathway and the cornea have been reported in diabetic animal models [5-7], but little is known about the effects of type 1 diabetes (T1D) on the limbus in rats or about the role of the OGF-OGFr pathway as a regulatory mechanism that becomes dysregulated in T1D. OGF, chemically termed [Met^5^]-enkephalin, is an endogenous peptide that inhibits cellular replication by upregulating cyclin-dependent inhibitory kinases p16 and p21 [10] and along with its receptor, OGFr, are present in the corneal epithelium across many species to maintain epithelial homeostasis [11-13]. Reports indicate that OGF levels are elevated in the serum of diabetic humans [14-17] and rats [8], as well as in the corneal epithelium [8,18]. Elevations of the inhibitory peptide OGF in diabetic animals are associated with ocular surface complications such as dry eye, abnormal corneal surface sensitivity, and delayed corneal epithelial repair [6,18-20], and may be the result of decreased cellular proliferation related to the elevated OGF levels. The magnitude of complications is sex-dependent and may have earlier onset and greater magnitude in female diabetic rats relative to male diabetic rats [18]. The onset of ocular surface complications is not related to hormonal differences as there is no correlation between estrogen or testosterone levels and OGF serum concentrations [21], although levels of both hormones are substantially reduced in hyperglycemic rats.

Blockade of the regulatory interactions between OGF and OGFr using naltrexone (NTX), a general opioid receptor antagonist, reverses dry eye [5,19] and enhances re-epithelialization of the corneal surface [20]. However, little is known about the relationships between the OGF-OGFr pathway, diabetes, and the limbus of male and female T1D rats. It is hypothesized that elevated OGF levels in the limbus of T1D animals may alter the overall morphology of the limbus. In this study, we evaluated whether the OGF-OGFr axis is dysregulated in the limbus of T1D male and female rats, and whether this abnormality is associated with altered limbal morphology (i.e., packing density, cell diameter), limbal stemness (i.e., CK15 expression), and limbal cell proliferation (i.e., Ki-67). In addition, we assessed mechanisms related to these defects by studying whether topical NTX can block the cellular changes observed in the limbus.

## Materials and Methods

### T1D rat model

All protocols were performed in accordance with the ARVO Statement for the Use of Animals in Ophthalmic and Vision Research and were approved by the Penn State College of Medicine Institutional Animal Care and Use Committee (protocol #47207). Seven independent experiments with small cohorts of male or female animals were conducted.

Male and female rats were included to ensure that sex was considered as a biological variable for vertebrate animal studies following NIH guidelines. Eight-week-old, adult male (101 -125 g body weight) or female (101-125 g body weight) Sprague Dawley rats (Charles River, Wilmington, MA) were acclimated to the facilities for one week prior to induction of hyperglycemia. All procedures followed previously reported protocols [5,6,18]. Rats were fasted for 6-12 hr before being injected intraperitoneally (i.p.) with 55 mg/kg streptozotocin (STZ) (Millipore-Sigma, MO) on two consecutive days. STZ was prepared by dissolving STZ powder in sodium citrate buffer (pH 4.5) at 4°C; the solution was prepared daily and used immediately. Rats had blood glucose levels >300 mg/dL within 72 hr and were designated as the diabetic group (T1D), and were randomly assigned to treatment groups. Animals of both sexes receiving i.p. injections of only sodium citrate buffer were considered controls.

### Physiological markers of T1D

Body weights and blood glucose levels were recorded at 4- and 8-weeks for a subgroup of male and female rats from each group.

### Mechanistic approach to therapeutic treatment of ocular surface complications by OGF-OGFr pathway blockade

Cohorts of male and female T1D rats were randomly selected to receive twice daily topical administration of NTX beginning on the first day of recorded hyperglycemia (=T1D_TopicalNTX_) or vehicle. Each rat received one drop (~50 μl) of 5 x 10^−5^ M NTX dissolved in phosphate-buffered saline and applied to the right cornea twice daily (0800 - 0900 hr and 1600 −1800 hr) following published procedures [6]. Animals did not require anesthesia for this procedure.

### Evaluation of ocular surface complications

To confirm that the OGF-OGFr pathway was dysregulated as reported previously, tear fluid volume and corneal epithelial sensitivity were measured periodically [6,17-20]. Tear volume was recorded using Schirmer tear strips (Alcon) placed in the lower lid cul-de-sac and sensitivity was measured using a Cochet-Bonnet aesthesiometer [5,19]. This latter test is expressed as g/mm^2^ such that an increased numerical score indicates that more force is required to elicit a blink response from the animal and therefore, decreased corneal sensitivity is noted.

At 4- and 8-weeks following confirmed hyperglycemia, randomly selected animals from each group and sex were humanely euthanized using a Euthanex Auto CO_2_ system attached to a smart box. The animal is placed in a standard box which allows carbon dioxide to be pumped into the box displacing oxygen at a rate of at least 30%. Within 2-3 minutes, when the rat has succumbed, cervical dislocation is used as a required second method of euthanasia following the carbon dioxide overdose. This procedure has been approved by the Penn State University College of Medicine IACUC committee for euthanasia of mice or rats. Eyes were removed from the orbital cavity and fixed in 4% PFA for 1 hour, followed by 30% sucrose and then embedded in OCT with isopentane chilled on dry ice and stored at −80°C until sectioned (14-18 μm) for immunohistochemistry or fixed in buffered formalin and processed in paraffin for morphological evaluation. Not all protocols were completed for both male and female rats at both time points due to COVID-19 restrictions and laboratory disruptions.

To confirm that the OGF-OGFr pathway was dysregulated in these animals, 4-8 eyes per sex per treatment group were processed for immunohistochemistry. Nonconsecutive sections were stained with antibodies to OGF (Penn State) (1:200) or OGFr (Abcam, ab251717) (1:200) following published procedures [5,6]. Sections stained with secondary antibody only (Alexa Fluor568 or 488) served as controls. OGF and OGFr immunostained sections were photographed with a Zeiss confocal microscope and R3Spot camera system. Densitometric measurements were made with NIH approved ImageJ optical density software. At least four sections/group/time from different rats were assessed in order to ensure rigor and reproducibility.

### Evaluation of the limbus morphology

Nonconsecutive 8 μm sections of the eye were stained with hematoxylin and eosin (H&E) to assess limbal cell diameter and packing density in the limbal epithelium using an Olympus BH-2 microscope equipped with an ocular grid. Cell packing density was determined by counting the number of cells in a grid area of 0.0064 μm^2^. Cell diameter was recorded at the widest dimension of approximately 20 cells/treatment at 40X magnification. Measurements were made from 4-6 sections per rat representing a minimum of 2 rats/sex/group at each timepoint. Two investigators examined the slides and were masked to the treatment group until recording the final data.

Evaluation of specific limbal cells was made following manufacturers’ instructions using antibodies to cytokeratin 15 (CK15; 1:200, MyBioSource, MBS300649) and published protocols [22]. Rates of cell proliferation were determined with anti-Ki67 antibodies (1:100, Abcam, ab15580), a known marker of cell replication. CK15 expression levels were quantitated using a Zeiss LSM 900 confocal microscope, and expression levels were measured using the NIH approved ImageJ Fiji optical density software [23].

Ki-67 staining was limited to evaluation of animals treated for only 4 weeks because of COVID restrictions on animal purchase, housing, and use. The number of total basal limbal cells within the entire limbus was counted based on DAPI staining. The number of Ki-67 positive basal limbal cells was counted and the ratio of proliferating to total limbal cells was calculated. Four- six sections were counted per group from multiple rats in order ensure rigor and reproducibility.

### Statistical analyses

Independent experiments were conducted requiring multiple shipments of male or female rats, ensuring rigor and reproducibility between the current experiments and published data. Data were examined using 1-way or 2-way ANOVA to evaluate male and female rats from all treatment groups at both time points. Subsequent comparisons were made using Newman-Keuls or Tukey tests. All data were analyzed with GraphPad Prism 8.0 software, and significance was established at p<0.05.

## Results

Seven independent experiments were conducted between May 2021 and July 2022 utilizing 30 female and 28 male rats; 1 female and 2 male animals died of hyperglycemia during that time period. The final experiment was conducted for only 4 weeks between August and November 2022.

### Overall T1D phenotype

The mean body weights of male and female rats at baseline were 180 ± 3 g and 155 ± 2 g, respectively. After 8 weeks, mean weights of the normal rats were 490 ± 25 g and 248 ± 5 g, for males and females, respectively. Hyperglycemic male and female rats weighed significantly less than their normal counterparts. At 4 weeks, male T1D rats weighed 300 ± 6 g and at 8 week weighed 320 ± 13 g, while female T1D rats weighed 207 ± 6 g at 4 weeks and 211 ± 3 g at 8 weeks. Topical NTX did not restore mean body weights of the T1D rats. The mean blood glucose levels for rats in all groups corresponded to mean values published previously [5,6]. Baseline blood glucose levels for male and female rats were 136 ± 12 mg/dL and 141 ± 10 mg/dL, respectively. Normal rats maintained their glucose levels over the 8-week period of time, whereas hyperglycemic animals had glucose levels greater than 500 mg/dL at both 4- and 8-week timepoints.

### Ocular surface defects in the cornea of T1D rats

Confirmation of a dysregulated OGF-OGFr axis was made through assessment of tear production and corneal surface sensitivity. Data from this study corresponded well with previously published values for mean tear volume and sensitivity in male and female T1D rats [5,6,18,21], demonstrating the reproducibility of the complications on the corneal surface following STZ-induced hyperglycemia. Corneal sensitivity measurements at baseline and at 4 and 8 weeks for normal male rats ranged from 0.39 to 0.40, whereas male T1D rats had sensitivity values of 0.49 ± 0.01 at 4 weeks and 0.87 ± 0.07 at 8 weeks, both values were significantly different from baseline at p<0.001. Topical NTX normalized the sensitivity scores such that at 4 and 8 weeks, values were 0.4 ± 0.01 and 0.49 ± 0.3, respectively. Sensitivity for rats in the male T1D_TopicalNTX_ group was significantly different (p<0.01) from baseline only at 8 weeks but differed (p<0.001) from that of T1D rats at both 4 and 8 weeks. Normal female rats had sensitivity scores of 0.4 throughout the 8-week period in comparison to T1D female rats with sensitivity values of 0.51 ± 0.01 and 0.8 ± 0.0 at 4 and 8 weeks, respectively, that were significantly elevated (abnormal values) relative to their own baseline. Rat eyes receiving NTX recorded sensitivity measurements at 4 weeks of 0.4 ± 0 and 0.52 ± 0.02 at 4 and 8 weeks.

Regarding tear production, the Schirmer scores for normal male and female rats ranged between 6 and 7 mm for male rats and between 6.8 and 7.2 mm for females. T1D female rats had Schirmer scores of 5.5 ± 0.25 mm at 4 weeks and 4.9 ± 0.1 mm at 8 weeks and were significantly reduced (p<0.05) from normal female values at both time points. NTX-treated T1D female rats had Schirmer scores in the treated eye that were 6.8 ± 0.4 mm and 6.6 ± 0.4 mm at 4 and 8 weeks, respectively, and were no longer considered “dry eye”. T1D male rats had Schirmer scores of 6.2 ± 0.2 mm at 4 weeks and 3.6 ± 0.2 mm at 8 weeks. NTX treatment reversed the dry eye and restored tear production to 6.7 ± 0.2 mm and 6.0 ± 0.4 mm at 4 and 8 weeks, respectively, with significant changes observed at 8 weeks only.

### Dysregulation of the OGF-OGFr pathway in the limbus

**Figure 1.**
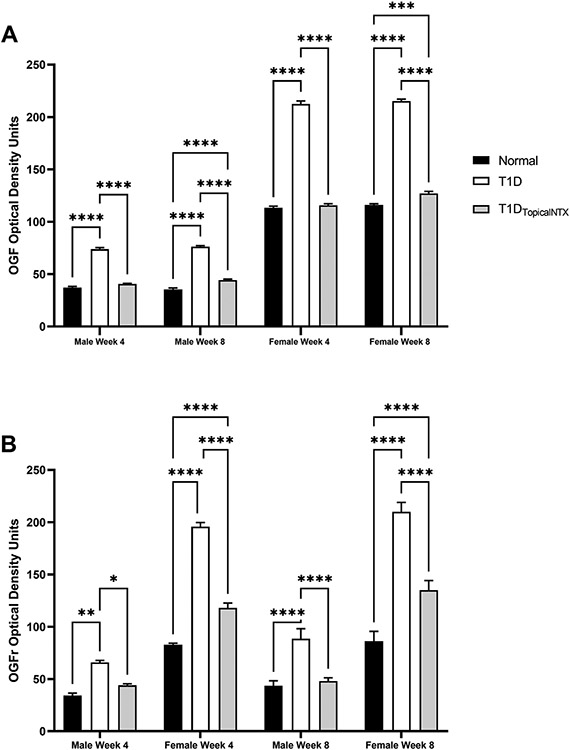
Quantitative assessment of OGF and OGFr expression in limbal tissues in normal and T1D males and female rats. Cohorts of male and female rats were treated with topical NTX twice daily for 4 or 8 weeks. Optical density values of OGF (A) and OGFr (B) expression in the limbal region are presented as mean + SEM. Significantly different from normal rats or T1D rats of the same sex at *p<0.05, **p<0.01, or ***p<0.001.

Additional evidence of the dysregulation of the OGF-OGFr pathway in the limbus came from assessment of expression patterns for the peptide and receptor (Figure 1). The immunohistochemical staining patterns of OGF and OGFr in the limbus were comparable to published images of limbus [5] and distribution of the OGF-OGFr pathway on the corneal epithelium [5,6]. Quantitative assessment of the optical density measurements of peptide and receptor were recorded across the basal layer of the limbal epithelium. Analyses of male data, F= 15.89 (df 6,72) p<0.001 indicated that optical density values of OGF staining ranged between 35 and 47 for male rats at 4 and 8 weeks in both normal and T1D_TopicalNTX_ groups, whereas T1D rats had elevated OD readings at both timepoints. Expression levels of OGFr were comparable to those previously published [6,18] and reflected elevated levels in the limbus for both male and female T1D rats relative to normals. Two-factor analysis (time, group) for female rats enabled further Bonferroni post-test analyses; F=190.3 (df 6,72) p<0.001. Female rats appear to have more OGF staining in the limbus than male counterparts, with female T1D animals recording approximately 200 OD units of OGF expression at 4 and 8 weeks in comparison to levels of approximately 100 OD units for normal and T1D_TopicalNTX_ female rats. Two-factor analysis of variance enabled further Bonferroni post-tests. T1D_TopicalNTX_ treatment resulted in normal OGFr levels within 4 weeks for male rats, and significant declines in OGFr expression from T1D levels were noted for female rats at both 4 and 8 weeks.

#### Limbal morphology:

**Figure 2.**
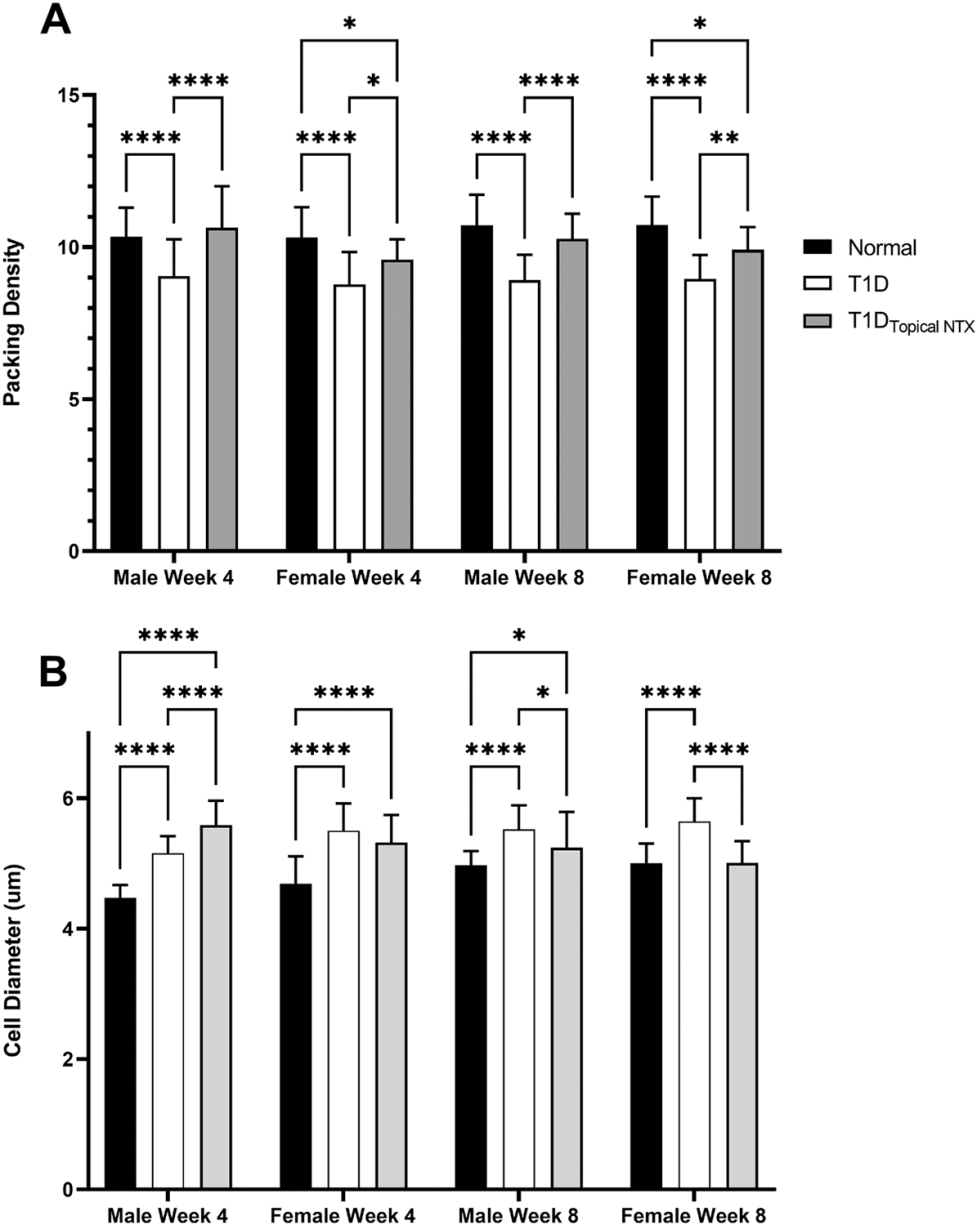
**(A)** Packing density (number of cells per mm^2^) and **(B)** cell diameter (mm) in the limbus of normal and T1D male and female rats. Cohorts of male and female rats were treated with topical NTX twice daily for 4 or 8 weeks. Values represent means ± SEM. Significantly different from normal or T1D rats of the same sex at *p<0.05, **p<0.01, or ****p<0.0001.

**Figure 3.**
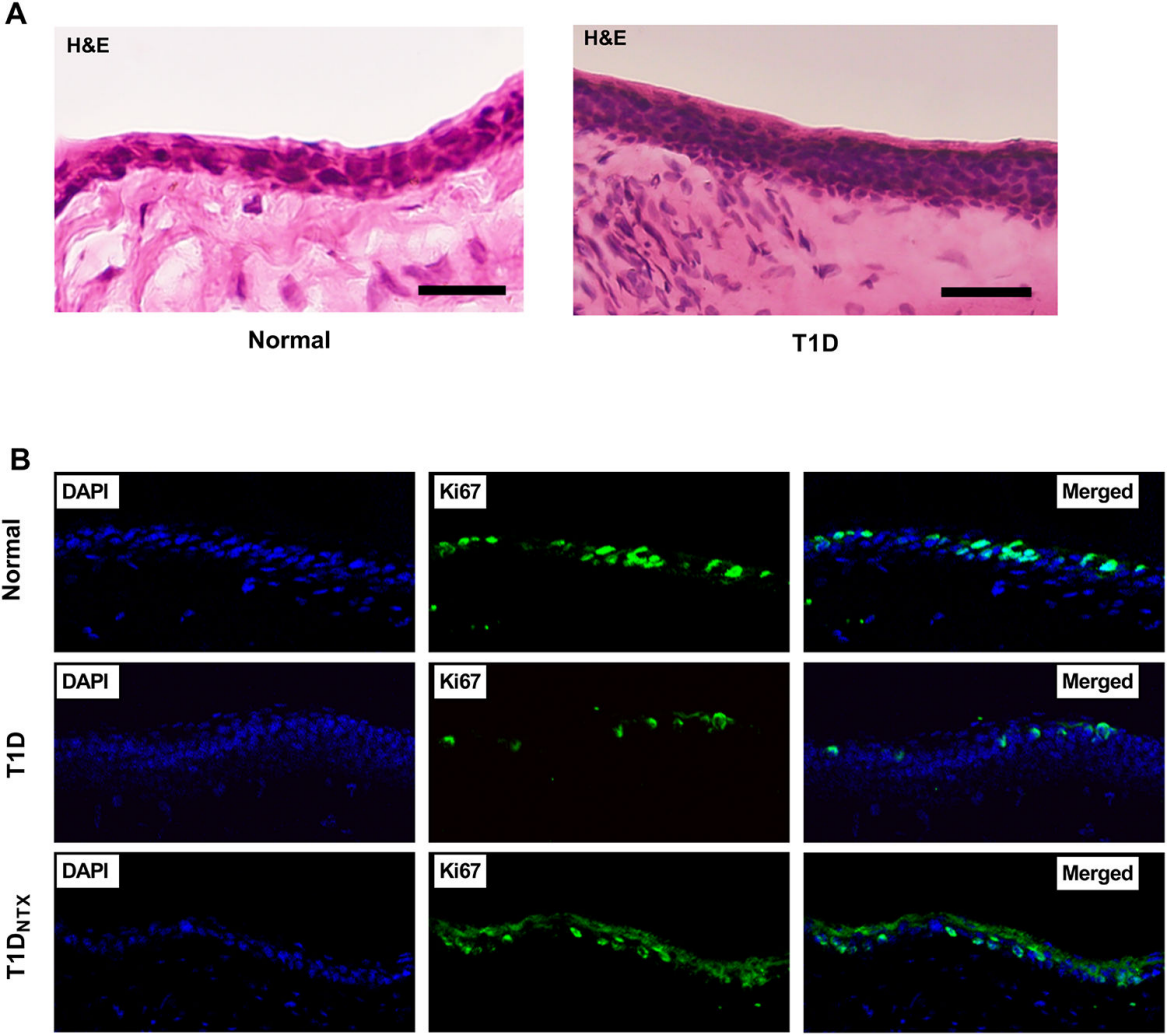
**(A)** Hematoxylin and eosin stained sections of the limbus of normal and T1D male rats. T1D male rats were hyperglycemic for 4 weeks. Images were captured at 40X. **(B)** Immunohistochemical staining of the comparable limbal sections of normal and T1D male rats. Some T1D rats were treated with topical NTX twice daily for 4 weeks (=T1D_NTX_). Images of DAPI, Ki67 and merged images are presented at 40X magnification.

**Figure 4.**
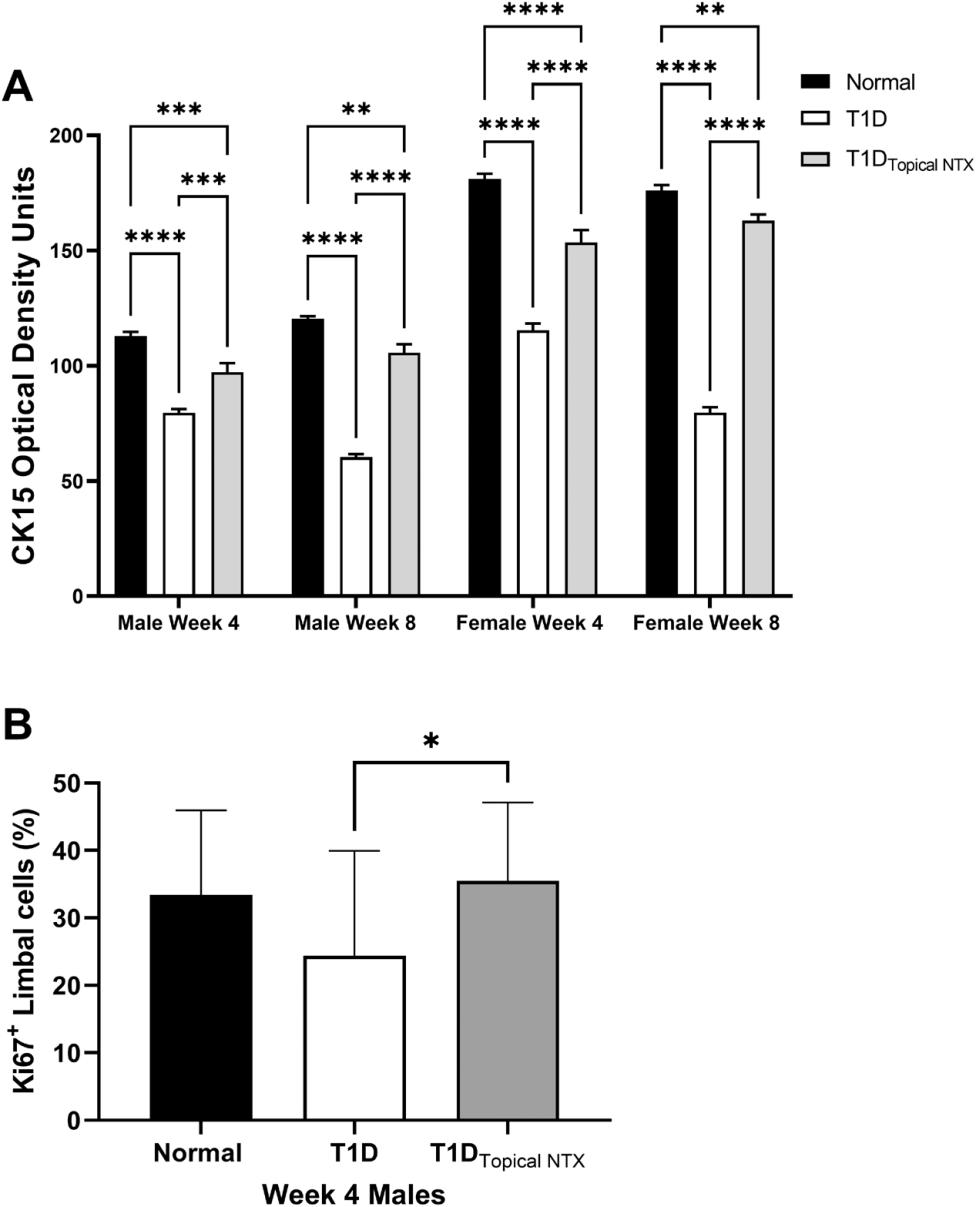
**(A)** CK15 expression (optical density) in the limbal region of normal and T1D male and female rats. Some cohorts of male and female T1D rats were treated with topical NTX twice daily for 4 or 8 weeks. Optical density measurements are presented as mean ± SEM. Significantly different at **p<0.01, ***p<0.001, and ****p<0.0001. **(B)** Ki67 positive limbal cells (percentage of total cells) were counted in the basal layer of the limbus of male rats after 4 weeks of treatment. Treatment groups included T1D rats treated with topical NTX twice daily, as well as normal and T1D male animals. Values represent means + SEM. Significantly different at *p <0.05.

The effect of hyperglycemia on the limbus is evaluated (Figures 2-4). Changes in cell diameter and packing density are showed in Figure 2 using hematoxylin and eosin stained sections (representative images shown in Figure 3A). The lower panel (Figure 3B) reveals the staining patterns for DAPI and Ki67 in male rats treated with NTX. Cell counts of CK15 and KI67+ limbal cells are presented in Figure 4A and 4B, respectively.

Epithelial thickness measurements in the limbus of normal male and female rats were ~4 μm, in comparison to measurements ranging from 4.5 to 6 μm in diabetic animals. Valid comparisons were difficult to make because of variability in the orientation and intactness of limbal sections and no further assessments of limbal thickness were made.

Packing density (number of cells/0.0015mm^2^) for male and female rats from each group at 4 and 8 weeks revealed that normal Sprague-Dawley rats had packing densities of 10.3 and 10.7 cells per area (Figure 2A). In comparison, male and female T1D animals had significantly (p<0.001) lower (gender and age-matched) packing density measurements with a packing density of 8.8 and 8.95 cells/0.0015 mm^2^ at 4 and 8 week, respectively, in female T1D rats, and a packing density of 9 and 8.9 cells/area for male T1D rats at 4 and 8 weeks, respectively. Treatment with NTX resulted in packing density measurements of 9.6 and 9.9 for female T1D rats receiving NTX at 4 and 8 weeks, respectively, and 10.6 and 10.3 cells/area for male rats T1D receiving NTX at 4 and 8 weeks, respectively.

Cell diameter of basal cells in the limbal epithelium ranged between 4.5 and 5.7 μM, with cells from T1D rats having a larger diameter than those in normal animals (Figure 2B) at both time points for male and female T1D rats. Cell diameter enlargement may contribute to the altered packing density.

#### Limbal cell stemness and proliferative capacity:

C K 1 5 expression as measured by optical density in the limbal region at weeks 4 and 8 in male and female animals is presented in Figure 4A. Within 4 weeks of STZ inoculations, CK15 levels for hyperglycemic male rats (75 OD units) were reduced more than 30% from that of normal males (~110 OD units) and 20% from male rats in the T1D_TopicalNTX_ group (~95 OD units). At 8 weeks, normal male rats displayed a comparable pattern to that at 4 weeks. T1D rats had even further reductions of CK15 relative to normal values (p<0.001) and the T1D_TopicalNTX_ group had levels significantly higher than T1D and no different than those measured in normal male animals. CK15 expression was greater in all groups of female rats relative to their respective treated male animals. CK15 levels in normal female animals were ~175 OD units at 4 and 8 weeks and were substantially higher than for male rats. T1D animals had significantly lower levels of CK15 at both time points suggesting fewer limbal stem cells. Optical density readings for female animals treated with NTX (i.e., T1D_TopicalNTX_ groups) had readings of ~155 OD units at both 4 and 8 weeks.

Figure 3B demonstrates the double-labeling of Ki67+ staining with DAPI. Figure 4B presents the mean number of replicating basal limbal cells counted in sections of eye from male diabetic and normal rats after 4 weeks of treatment twice daily with NTX. Data are limited to 4-week treatment for both sexes. The mean percentage of Ki67+ cells for normal male rats was 33.4 ± 0.04% and 35.0 ± 0.04% for T1D rats treated with NTX for 4 weeks. Male T1D rats receiving saline had mean proliferation indices of 24.7 ± 0.05%, a decrease of 26% from normal values.

## Discussion

Collectively, these data establish that the OGF-OGFr axis is present in the limbus of male and female rats and becomes dysregulated in the limbal region upon induction of hyperglycemia. Previous research has shown that dysregulation of the OGF-OGFr axis coincides with ocular surface complications [5,6,18]. Because a key role of the limbus is to provide epithelial cells that terminally differentiate and populate the corneal epithelium [8,9,22], we hypothesized that dysregulation of the OGF-OGFr axis in the limbus may impact limbal morphology that may in turn exacerbate the corneal surface complications associated with diabetes. *In vitro* models of human and rabbit corneal epithelial cells revealed the best outgrowth with serum that was rich in growth factors including CK15, CK3, ZO-1 and other integrins [24]. Human studies on limbal stem cells focus on how the limbus is important for epithelial wound healing [25-27]. Limbal epithelial stem cells on the diabetic ocular surface have been shown to be dysfunctional due to the numerous markers that are significantly diminished such as CK15 or Np63alpha among others [25,26]. A study on rabbit eyes has shown that CK15 expression is greatest in the limbus for control animals [26]. Expression of CK15 was decreased in diabetic models compared to the animal models [26]. In terms of dysfunctional limbal epithelial stem cells, experiments have been performed in which diabetic rats were injected with human hematopoietic stem cells resulting in the reversal of corneal epithelial changes [28].

The two markers examined in this study indicated that although they are important indicators of limbal homeostasis, both were altered by the elevated levels of OGF recorded on the limbal surface. This work demonstrates diminished Ki67 proliferation index and decreased CK15 expression in the limbal region of the ocular surface for T1D male and reduced CK15 expression in T1D female rats. Epithelial thickness of the cornea in diabetic models has been observed to be abnormal in numerous studies, but studies in the limbus are limited. Mean epithelial thickness in the limbus is elevated in diabetic animals and corresponds to the decreased packing density in the diabetic model [5,11,12]. The decreased packing density could be a result of fewer proliferating cells.

Topical NTX improves the expression of Ki67 and CK15 in the limbal region and could explain the increased and restored packing density in the ocular surface. This suggests that hyperglycemia has a direct effect on the limbus resulting in decreased Ki67 and CK15 expression, thereby leading to abnormal epithelial thickness and packing density in the limbal region. These observations were evident in both the male and female rats. The magnitude of complications is elevated in the female model compared to the male model. In previous studies, the elevations of serum OGF in diabetic models were postulated to contribute to the elevation of OGF expression reported in the cornea. This study suggests that the elevation of OGF expression in the limbus could elicit ocular surface complications as a result of OGF dysregulation within the limbus.

These data support the hypothesis that the corneal surface dysregulation in diabetes could be a result of dysregulation of the OGF-OGFr axis in the limbus that is partially insulin independent. All of the mechanisms contributing to the altered limbal morphology in the diabetic rat are not known, but may include systemic inflammation caused by diabetes reducing cell replication, as well as deficiencies in insulin protection [29]. Dry eye and abnormal corneal surface sensitivity are still apparent in T1D rats that are euglycemic with insulin minipumps [29]. However, corneal epithelial wound healing abnormalities in T1D rats that involve cell replication do appear to be responsive to insulin in that rates of wound closure are comparable to those of normal rats [29]. Collectively these data suggest that the OGF-OGFr axis impacts corneal surface homeostasis in diabetes in a manner that is at least partially insulin independent and that a better understanding of the role of the limbal OGF-OGFr axis in ocular surface complications in diabetes could be informative regarding the pathophysiology of T1D complications and lead to improved therapies.
